# Emerging roles of glutaredoxins in plant metabolism

**DOI:** 10.1093/jxb/erag201

**Published:** 2026-04-27

**Authors:** Anna Moseler, Jean-Philippe Reichheld, Andreas J Meyer

**Affiliations:** INRES-Chemical Signalling, University of Bonn, Bonn D-53117, Germany; Laboratoire Génome et Développement des Plantes, Université Perpignan Via Domitia, Perpignan F-66860, France; Laboratoire Génome et Développement des Plantes, CNRS, Perpignan F-66860, France; INRES-Chemical Signalling, University of Bonn, Bonn D-53117, Germany; Michigan State University, USA

**Keywords:** Development, foldase, glutaredoxin, glutathione, holdase, iron-sulfur cluster, metabolism, oxidoreductase, transcription factor

## Abstract

Glutaredoxins (GRXs) constitute a distinct family within the large thioredoxin superfamily. Based on the historic order of discoveries and structural similarities, GRXs were originally annotated as glutathione-dependent oxidoreductases with a CxxC active site motif. These prototypical GRXs are now clustered as class I. Later, it was discovered that some GRXs, particularly those with the highly conserved active site motif CGFS, coordinate iron-sulfur clusters and exhibit little or no oxidoreductase activity. Throughout the course of evolution across kingdoms, the CGFS-containing GRXs form the most highly conserved class II cluster. Compared with non-plant species, the GRX family in spermatophytes has undergone significant enlargement and diversification, resulting in typically more than 30 isoforms that are separated into four classes. This expansion is largely due to the evolution of new plant-specific class III GRXs or ROXYs, characterized by a central CCxx active site motif. The increasing number of *GRX* copies in the genome, coupled with the modification of domains and addition of domains with different functions, has resulted in the functional diversification of plant GRXs. In this review, we aim to provide an overview of the established and emerging roles of GRXs in plant metabolism.

## Teaser

The expanded family of plant glutaredoxins is structurally similar, yet shows functional diversification, including redox-dependent modification of protein thiols, transfer of iron-sulfur clusters, support of protein folding, and transcriptional control.

## Introduction

Glutaredoxins (GRXs) are small thiol proteins which belong to the thioredoxin (TRX) superfamily based on their structure. The TRX superfamily is characterized by a spatial topology, consisting of a four-stranded antiparallel β-sheet surrounded by three α-helices (β1-α1-β2-α2-β3-β4-α3). The first α-helix contains a four-amino acid active site motif, which includes one or more cysteines (Eklund *et al*., 1992; Xia *et al*., 1992). GRXs were first discovered 50 years ago by Arne Holmgren as a hydrogen donor system for *Escherichia coli* ribonucleoside-diphosphate reductase dependent upon reduced glutathione (GSH) (Holmgren, 1976). The first plant GRXs were identified in rice and spinach only twenty years later. GSH-dependent thiol transferase activities in these cases were confirmed with L-cystine or bis(2-hydroxyethyl) disulfide (HED) as artificial substrates (Minakuchi *et al*., 1994; Morell *et al*., 1995).

The release of the *Arabidopsis thaliana* genome in 2000 revealed that the GRX family is far more complex in plants than in non-plant species. While the GRX family comprises only five members in human and eight isoforms in *Saccharomyces cerevisiae*, the Arabidopsis genome encodes 33 GRXs and GRX-like proteins (Fig. 1). These proteins are classified into four classes according to their primary sequence: six belong to class I, four to class II, 21 to class III, and two to class IV. The class IV isoforms are annotated as GRX-like proteins because they contain a GRX-like domain fused with other domains of unknown function. While more than 30 GRX isoforms are typical of diploid land plants (Couturier *et al*., 2009a), larger numbers of up to 157 GRXs have been reported in some species, with *Hibiscus syriacus* having the largest known number (Garg *et al*., 2010; Wang *et al*., 2023; El Baidouri *et al*., 2024). However, these genomic analyses include another distantly related protein family of GRX-like proteins, which are characterized by four conserved CxxC motifs (Navrot *et al*., 2006). Due to the current lack of functional information and the fact that these proteins are only distantly related to the GRX family, they are not considered further here.

**Fig. 1.**
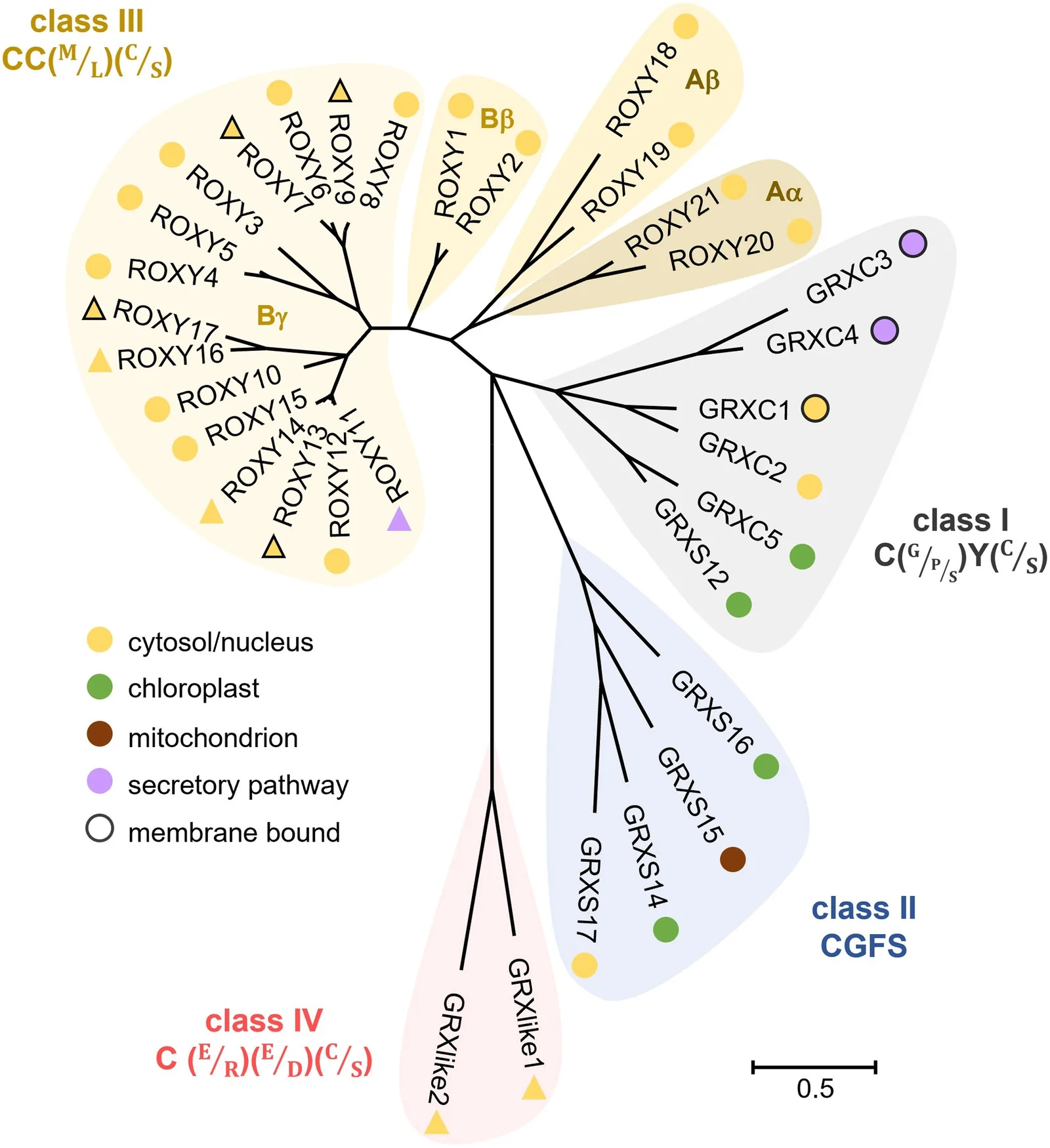
Phylogenetic analysis of Arabidopsis glutaredoxins. An unrooted tree calculated on aligned Arabidopsis glutaredoxins using the full-length amino acid sequences is shown. The evolutionary history was inferred by using the Maximum Likelihood method and JTT matrix-based model in MEGA11 (Tamura *et al*., 2021). The scale bar represents 0.5 substitutions per amino acid position. The dots indicate experimentally confirmed sub-cellular localizations. Triangles indicate predicted localization based on SUBA5 (Hooper *et al*., 2017). For further description of membrane localization of class I GRXs see Fig. 2 and the main text. The nomenclature for class I and class II GRXs follows the designation proposed by Rouhier *et al*. (2004).Class III GRXs are named ROXYs here, after the first isoform of this class that was identified in a genetic screen (Xing *et al*., 2005). The class III has already split into two clades, A and B, each of which has two sub-groups, Aα and Aβ and Bβ and Bγ, respectively. Accession numbers: GRXC1 (At5g63030), GRXC2 (At5g40370), GRXC3 (At1g77370), GRXC4 (At5g20500), GRXC5 (At4g28730), GRXS12 (At2g20270), GRXS14 (At3g54900), GRXS15 (At3g15660), GRXS16 (At2g38270), GRXS17 (At4g04950), GRXC6 (ROXY21, At4g33040), GRXC7 (ROXY1, At3g02000), GRXC8 (ROXY2, At5g14070), GRXC9 (ROXY19, At1g28480), GRXC10 (ROXY20, At5g11930), GRXC11 (ROXY4, At3g62950), GRXC12 (ROXY5, At2g47870), GRXC13 (ROXY9/CEPD2, At2g47880), GRXC14 (ROXY8/CEPDL2, At3g62960), GRXS1 (ROXY16, At1g03020), GRXS2 (ROXY10, At5g18600), GRXS3 (ROXY11, At4g15700), GRXS4 (ROXY13, At4g15680), GRXS5 (ROXY12, At4g15690), GRXS6 (ROXY17, At3g62930), GRXS7 (ROXY14, At4g15670), GRXS8 (ROXY15, At4g15660), GRXS9 (ROXY7/CEPDL1, At2g30540), GRXS10 (ROXY3, At3g21460), GRXS11 (ROXY6/CEPD1, At1g06830), GRXS13 (ROXY18, At1g03850), GRXlike1 (At4g08550), GRXlike2 (At3g11920).

Class I and class II GRXs are widespread amongst both prokaryotes and eukaryotes. The highly conserved active site motifs C(G/P/S)Y(C/S) in class I and CGFS in class II, suggest that they play fundamental roles in cellular processes. In contrast, class III, which is defined by an active site of CC(M/L)(C/S) and some further, more rarely used substitutions, is exclusive to the plant kingdom, with low numbers found in bryophytes and a pronounced diversification in spermatophytes, particularly in angiosperms (Xing *et al*., 2006). Based on a reduced number of petals as the first distinct phenotype for *grx* mutants in Arabidopsis, class III GRXs have been termed ROXYs (Xing *et al*., 2005). The fourth class is present in photosynthetic eukaryotes, and includes GRX-like proteins with an N-terminal GRX domain having a CEEC and CRDS active site motif, respectively, followed by two domains of unknown function (Couturier *et al*., 2009a). Please note that the variations indicated here for active site motifs only reflect diversity in Arabidopsis. In other plant species, the variable positions may show other amino acids, contributing to the large diversity and likely adaptive evolution of the GRX systems (Wang *et al*., 2023).

Classical TRXs act as oxidoreductases that activate or deactivate target proteins by thiol-disulfide exchange reactions. The catalytic mechanism for the reduction of target proteins involves a nucleophilic attack of a reduced TRX with its active site cysteine on a disulfide of the respective target protein and the formation of a mixed disulfide intermediate of both proteins, which is subsequently resolved by a nucleophilic attack of the second resolving TRX thiol moiety, resulting in the release of a disulfide-containing TRX (Brandes *et al*., 1993). This feature has subsequently been broadly exploited for capture and identification of TRX targets by substituting the resolving cysteine, which causes stabilization of the mixed disulfide intermediate and enables their isolation (Brandes *et al*., 1996; Verdoucq *et al*., 1999; Balmer and Schürmann, 2001; Motohashi *et al*., 2001). In contrast, GRXs do not form mixed disulfides with their target proteins. This means that a similar fishing approach cannot be used, which has severely delayed their functional analysis. In this case, the putative target proteins can only be identified individually in time-consuming biochemical assays. An additional challenge for the functional analysis of plant GRXs is the high degree of functional redundancy resulting from multiple rounds of genome duplications. In consequence, single deletion mutants generated in reverse genetics approaches often do not result in obvious phenotypes. During the first two decades of research into *GRX* genes in plants, only three such genes in Arabidopsis (*ROXY1*, *GRXS15* and *GRXS17*) were found to result in strong phenotypes when deleted (Xing *et al*., 2005; Cheng *et al*., 2011; Moseler *et al*., 2015). Meanwhile, more subtle phenotypes have been reported for several other deletion mutants exposed to abiotic or biotic stress factors (La Camera *et al*., 2011; Laporte *et al*., 2012; Rey *et al*., 2017).

This review aims to provide an overview of the current knowledge on the roles of members of the different GRX classes in plants, focusing particularly on their emerging roles in plant metabolism. Because most genetic evidence is available for Arabidopsis, we use the corresponding Arabidopsis gene set as a core to structure the review, and include information from other species as appropriate.

## Class I glutaredoxins function as redox regulators

Arabidopsis class I GRXs comprises six members called GRXC1-5 and GRXS12, with a canonical C(G/P/S)Y(C/S) active site motif. Their phylogenetic separation into three subgroups (Fig. 1) correlates with their subcellular localization to cytosol, endoplasmic reticulum (ER) and chloroplasts (Fig. 2A) (Couturier *et al*., 2011; Schlößer *et al*., 2024). Interestingly, no class I GRXs are present in peroxisomes and mitochondria, even though both compartments are known hotspots for the formation of reactive oxygen species (ROS), and do contain glutathione disulfide reductases for maintenance of a highly negative glutathione redox potential (*E*_GSH_) (Schwarzländer *et al*., 2008; Marty *et al*., 2019). Although class I GRXs are broadly conserved across all kingdoms, plants acquired new isoforms which are present in chloroplasts. Most cyanobacteria contain one class I GRX isoform, which differ significantly from eukaryotic class I GRXs in their primary sequence (Couturier *et al*., 2009a). Hence, it can be assumed that higher plants obtained their plastidic GRXs from cytosolic precursors, rather than from the cyanobacterium during endosymbiosis. Additionally, two isoforms are present in each respective subcellular compartment of Arabidopsis, raising questions about their role, and of how plant-specific physiology has shaped GRX evolution. Class I GRXs have a distinct oxidoreductase activity, which allows the reduction of dehydroascorbate (DHA), type II peroxiredoxins, and methionine sulfoxide reductases (Couturier *et al*., 2011, 2013; Vogelsang and Dietz, 2023). The use of redox-sensitive GFP (roGFP) as an artificial model substrate protein has revealed that GRXs not only reduce substrates, but rather, also glutathionylate substrate proteins (Meyer *et al*., 2007; Trnka *et al*., 2020; Geissel *et al*., 2024), which suggests GRXs can act bidirectionally in stress-related oxidative responses and subsequent recovery processes.

**Fig. 2.**
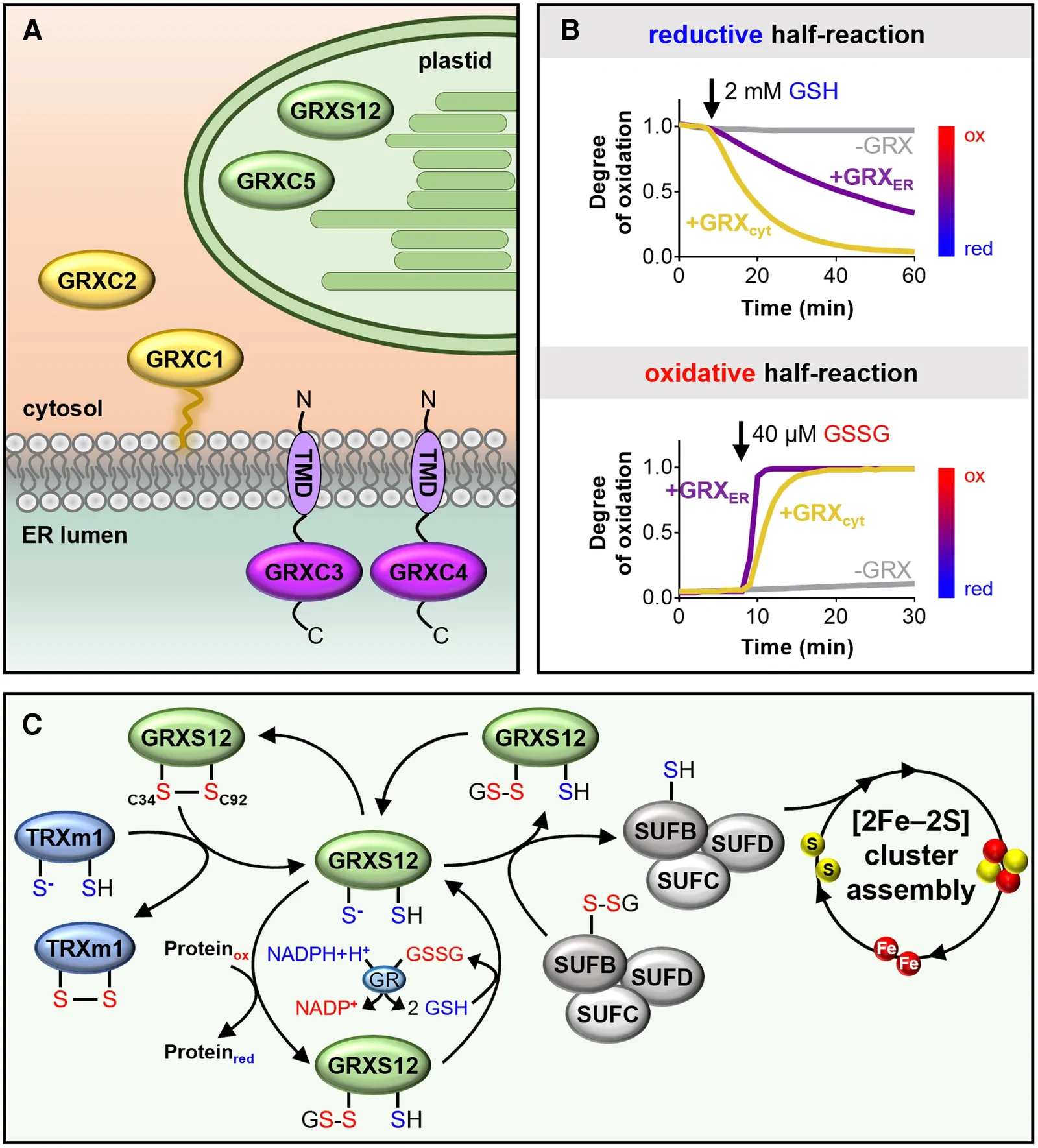
Sub-cellular localization and features of class I glutaredoxins. (A) Sub-cellular localization of class I GRXs. GRXC1 and GRXC2 are localized to the cytosol with GRXC1 being attached to the endomembrane system by myristoylation. GRXC3 and GRXC4 are type II transmembrane proteins with their N-termini facing the cytosol and the C-termini including the GRX domain present in the ER lumen. GRXC5 and GRXS12 are present in the plastids. (B) Oxidoreductase activity of class I GRXs. Interaction assays with roGFP2 as a redox-dependent fluorescent target protein enables direct monitoring of disulfide reduction with GSH as electron donor, and disulfide formation with GSSG as electron acceptor. ER luminal and cytosolic GRXs differ in their kinetic properties, with cytosolic GRXs being better catalysers for the reductive half-reaction, and ER luminal GRXs showing enhanced catalytic properties in the oxidative half reaction. The figure shows a graphical illustration of experimental data reported by Schlößer *et al*. (2024; redrawn with permission). (C) Model for the GRXS12-mediated redox regulation of SUFB and other chloroplast proteins as proposed by Liu *et al*. (2026; redrawn with permission). The oxidoreductase activity of GRXS12 depends on the active site C^34^ and can directly reduce SUFB, thereby regulating Fe–S cluster assembly. C^34^ can undergo glutathionylation, or form an intramolecular disulfide bond with C^92^. The subsequent recycling of the oxidized residues occurs either via the GSH/GR system or TRXm1, respectively. The unnamed redox-active thiol proteins (Protein_ox_ or Protein_red_) might be involved in multiple metabolic and signaling processes. SUF: sulfur utilization factor; GR: glutathione disulfide reductase. Blue color indicates reduced thiols and red indicates oxidized thiols.

### Cytosolic GRXC1 and GRXC2

GRXC1 and GRXC2 are both cytosolic, with GRXC1 being attached to the endomembrane system and the plasma membrane through N-terminal myristoylation (Riondet *et al*., 2012; Schlößer *et al*., 2024). *In vitro*, both enzymes are able to efficiently reduce or oxidize artificial substrates such as HED or roGFP2 in a glutathione-dependent manner (Vogelsang and Dietz, 2023; Schlößer *et al*., 2024). Furthermore, *in vitro* studies showed that both proteins play a role in the regulation/regeneration of thiol-dependent enzymes. Both GRXC1 and GRXC2, are able to reduce the peroxiredoxin PRXIIB and MALATE DEHYDROGENASE 1 (Brehelin *et al*., 2003; Vogelsang and Dietz, 2023), and to deglutathionylate the cytosolic TRIOSEPHOSPHATE ISOMERASE (Dumont *et al*., 2016). Further studies showed that GRXC1 can also reduce TRXh3 (Reichheld *et al*., 2007) and a persulfide (-SSH) on the 3-MERCAPTOPYRUVATE SULFURTRANSFERASE 2 (Moseler *et al*., 2021a). GRXC2 is able to glutathionylate the BRI-ASSOCIATED RECEPTOR-LIKE KINASE 1 (Bender *et al*., 2015). Despite robust *in vitro* data indicating that they act as glutathione-dependent oxidoreductases, the roles of GRXC1 and GRXC2 *in planta* remain less clearly defined. Phenotypes of *grxc1* or *grxc2* null mutants are absent under normal growth conditions or stress conditions such as high temperature, osmotic stress caused by mannitol, or reductive stress induced by dithiothreitol (Riondet *et al*., 2012; Dard *et al*., 2023; Schlößer *et al*., 2024). For oxidative stress, concentrations of 1 mM or higher hydrogen peroxide (H_2_O_2_) did not cause a phenotype, while lower H_2_O_2_ concentration of 100 µM led to reduced root growth of a *grxc1* mutant (Riondet *et al*., 2012; Zhang *et al*., 2025). The *grxc2* mutant was, however, not tested with lower H_2_O_2_ concentrations. Even though redundancy would be expected based on localization and biochemical properties, this might be rather unlikely, because *grxc1 grxc2* double mutants are viable and do not exhibit any enhanced phenotypes (Schlößer *et al*., 2024). A putative redundancy with the TRX system cannot be excluded based on electrostatic complementarity (Bodnar *et al*., 2023). Functional redundancy between GRX and TRX systems was also apparent from early work in yeast in which it was shown that a single *GRX* or *TRX* gene is essential for viability (Draculic *et al*., 2000). More recently, it was shown that a single monothiol class I GRX isoform is sufficient to replace all cytosolic TRXs and dithiol class I GRXs in yeast (Zimmermann *et al*., 2020).

A closer examination of the two cytosolic GRXs in Arabidopsis indeed reveals subtle differences, which may indicate specific functions. The most striking feature is that GRXC1 is specific to eudicots, implying that it may have evolved in parallel with unique characteristics of this plant group (Couturier *et al*., 2009a). Furthermore, GRXC1, which has a CGYC active site motif, is able to form a dimer and coordinate a [2Fe–2S]-cluster. Because the cluster ligands include the first active site cysteines of both GRX monomers and two GSH molecules, the cluster coordinating GRXC1 dimer is enzymatically inactive. In contrast, GRXC2 with a CPYC active site motif is not able to coordinate a Fe–S cluster, which is in line with the absence of the Fe–S cluster in a GRXC1 variant having a G/P substitution, indicating that proline prevents the cluster coordination (Rouhier *et al*., 2007; Riondet *et al*., 2012). GRXC1 was also found as a dimer *in planta* (Riondet *et al*., 2012), but the presence of Fe–S cluster and the functional link between Fe–S cluster binding and redox regulation *in vivo* remains to be fully elucidated.

As mentioned before, GRXC1 is bound to membranes with an N-terminal myristoyl group (Schlößer *et al*., 2024). Although myristoylation is necessary for membrane targeting, the weak binding energy provided by the myristate moiety is insufficient for stable membrane anchoring, which in turn could indicate that GRXC1 and GRXC2 can target the same proteins (Majeran *et al*., 2018; Meinnel *et al*., 2020). Nevertheless, there are supposed functions of GRXC1 in which localization at the membrane is relevant (Baune *et al*., 2020; Zhang *et al*., 2025). GRXC1 was identified as an interaction partner of the GLUCOSE-6-PHOSPHATE (G6P)/PHOSPHATE TRANSLOCATOR isoform, GPT1 (Baune *et al*., 2020). GPT1 is a membrane protein dually targeted to peroxisomes via the ER and to the inner envelope membrane of plastids, and imports glucose-6-phosphate (G6P) to fuel the oxidative pentose-phosphate pathway (OPPP). This pathway involves several steps, in which G6P is converted into ribulose-5-phosphate (Ru5P), generating reduction power in form of two molecules of NADPH (reviewed in Kruger and von Schaewen (2003)). Although the full pathway is only found in plastids, some OPPP reactions also occur in the cytosol and peroxisomes (Hölscher *et al*., 2016; Doering *et al*., 2024; Linnenbrügger *et al*., 2025). By investigating how enzymes involved in the OPPP are re-directed from plastids or the cytosol to peroxisomes, it was found that GPT1 interacts with GRXC1 at the ER and its substructures, but not in plastids (Baune *et al*., 2020). It was suggested that GRXC1 facilitates the insertion of GPT1 into the ER membrane and/or its sorting to peroxisomes. The precise mechanism, however, was not described. Notably, two translocators are present in Arabidopsis, namely GPT1 and GPT2 (Niewiadomski *et al*., 2005). An interaction with GRXC1 was found with GPT1, but not with GPT2. This is of interest, because GPT1 is solely present in eudicots, similar to GRXC1 (Baune *et al*., 2020). Hence, it would be interesting to analyse whether the localization of GPT1 is affected in *grxc1* null mutants to validate the importance of GRXC1 in this process.

Another interaction of GRXC1 was shown for the plasma membrane-bound receptor CANNOT RESPOND TO DMBQ 1 (CARD1), also known as HYDROGEN-PEROXIDE-INDUCED Ca^2+^ INCREASES 1 (HPCA1) (Zhang *et al*., 2025). CARD1 is a leucine-rich repeat receptor-like kinase highly conserved in land plants that contains an apoplastic domain and an intracellular kinase domain. It was identified as an extracellular sensor of H_2_O_2_ and quinones, and plays a role in plant acclimation to stress by triggering the accumulation of H_2_O_2_ and calcium as part of a signaling mechanism in the cytosol (Laohavisit *et al*., 2020; Wu *et al*., 2020; Fichman *et al*., 2022). By analysing the downstream targets and regulation mode of CARD1, GRXC1 was identified as an interaction partner of CARD1 (Zhang *et al*., 2025). While no interaction of GRXC2 and CARD1 was detected, interaction of CARD1 and GRXC1 was exclusively detected at the plasma membrane, with GRXC1 being present as a dimer. Upon treatment with the quinone, 2,6-dimethoxy-1,4-benzoquinone (DMBQ), cytosolic H_2_O_2_ increased, and GRXC1 was oxidized at its catalytic cysteine C^39^. After DMBQ treatment, a reduced amount of dimeric GRXC1 was found to interact with CARD1 (Zhang *et al*., 2025). It is tempting to speculate that oxidation of C^39^ promotes loss of the Fe–S cluster and subsequent monomer formation. Previous *in vitro* studies have shown that GRXC1 gradually loses its Fe–S cluster when oxidants, including H_2_O_2_, are added (Riondet *et al*., 2012). Because of the vulnerability to oxidative conditions, coordination of the Fe–S cluster was previously suggested to serve as a redox sensor for GRX activation (Lillig *et al*., 2005). Consistent with its kinase activity, CARD1 was able to phosphorylate GRXC1 at the threonine T^55^. While a phosphomimetic T^55^D variant interacted only weakly with CARD1, the non-phosphorylatable T^55^A variant was able to interact with CARD1. This indicates that the phosphorylation level of GRXC1 impacts the strength of its interaction with CARD1. Additionally, substitution of the redox-sensitive C^39^ residue by a serine diminished GRXC1 phosphorylation, implicating the redox status in controlling phosphorylation. These findings point to a common DMBQ–CARD1–GRXC1 signaling module, which was further emphasized by attenuated root growth inhibition upon DMBQ exposure in the *card1* and *grxc1* null mutants (Zhang *et al*., 2025). Nevertheless, the precise function of GRXC1 and physiological targets still remain largely unknown.

### ER luminal GRXC3 and GRXC4

GRXC3 and GRXC4 were both found to be type II transmembrane proteins in the early secretory pathway. Both GRXs contain a single-spanning transmembrane domain of 22 and 21 amino acids, respectively, in their N-terminal region. This results in the N-termini being oriented towards the cytosol, while the C-termini, including the GRX domains, are oriented towards the ER lumen (Schlößer *et al*., 2024). Similar to GRXC1 and GRXC2, GRXC3 and GRXC4 are able to reduce or oxidize artificial substrates like HED or roGFP2 in a glutathione-dependent manner (Schlößer *et al*., 2024). Previous biochemical analysis of poplar GRXC3 and GRXC4 indicated a separation of cytosolic and luminal GRXs with reference to their catalytic properties. Using HED and DHA assays, cytosolic GRXC1 and GRXC2 appeared to be less efficient catalysts compared with luminal GRXC3 and GRXC4 (Couturier *et al*., 2013). Similar decreased activity of cytosolic GRXC1 and GRXC2, compared with luminal GRXC3 and GRXC4, was observed for Arabidopsis GRXs in the HED assay (Schlößer *et al*., 2024). Analysing both the reducing and oxidizing capacities with roGFP2 as an alternative substrate, GRXC1 and GRXC2 catalysed the reduction of oxidized roGFP2 faster than GRXC3 and GRXC4, which suggests that catalytic efficiency of GRXs in reductive reactions depends on the corresponding substrate (Fig. 2B). Conversely, GRXC3 and GRXC4 exhibited enhanced oxidizing capabilities for roGFP2 compared with GRXC1 and GRXC2 (Fig. 2B), suggesting an adaptation to the different redox conditions in each compartment (Schlößer *et al*., 2024). Beside the biochemical and subcellular differentiation of cytosolic and luminal GRXs, the formation of the subgroups is further supported by a separation based on their electrostatic properties (Bodnar *et al*., 2023; Schlößer *et al*., 2024). Similar to GRXC1 and GRXC2, however, the physiological targets and precise function of GRXC3 and GRXC4 remain to be explored. A plausible hypothesis is that luminal GRXs are involved in oxidative protein folding by supporting the reduction of falsely formed disulfides and the formation of correct disulfides (Meyer *et al*., 2021). These processes would involve intermittent glutathionylation and deglutathionylation of target proteins.

One striking structural difference is a serine instead of cysteine in GRXC3/4 compared with GRXC1/2 is the first amino acid of α-helix 3, which is in close proximity to α-helix 1 carrying the active site motif. This residue has been shown to affect the biochemical properties of class II GRXs (Zaffagnini *et al*., 2008; Zannini *et al*., 2019). Its relevance for the biochemical properties of GRXC3/4 has not been investigated, but will be discussed in the context of class II GRX later in this review. Single and double null mutants for *GRXC3* and *GRXC4* showed no obvious developmental phenotype and were not sensitive towards high temperature, osmotic stress, and reductive stress (Dard *et al*., 2023; Schlößer *et al*., 2024). Because both ER luminal GRXs contain a CPYC active site motif and originated from a gene duplication in euphyllophytes during or shortly after their separation from lycophytes (Couturier *et al*., 2009a), functional redundancy is likely. However, it is difficult to assess whether redundancy actually exists, as a double null mutant does not exhibit a macroscopically visible phenotype (Schlößer *et al*., 2024). This raises the further question of the physiological conditions under which these luminal GRXs become essential, such as developmental stage, stress, and tissue.

### Plastidic GRXC5 and GRXS12

In Arabidopsis, GRXC5 and GRXS12 are found in the chloroplast stroma (Couturier *et al*., 2011). Unlike the other subgroups, which both contain two dithiol GRXs, the plastidic subgroup comprises the dithiol GRXC5, which has a CSYC active site motif, and the monothiol GRXS12, which has a CSYS motif. These two isoforms only occur together in species of the *Brassicaceae* family, while other species contain either GRXC5 (e.g. *Physcomitrium patens* and *Medicago truncatula*) or GRXS12 (e.g. poplar and rice) (Couturier *et al*., 2009a; Müller-Schüssele *et al*., 2021). Of both Arabidopsis proteins, only GRXC5 can bind an Fe–S cluster that is coordinated with the cysteine ligands from two GRXs and two GSH molecules, analogous to the cluster coordination of GRXC1 (Couturier *et al*., 2011). Plastidic GRXs are active in the standard assays using artificial substrates such as HED or DHA (Couturier *et al*., 2009b, 2011; Zaffagnini *et al*., 2012). Additionally, *P. patens* GRXC5 was shown to efficiently reduce and oxidize roGFP2. Direct comparison with Arabidopsis GRXC1 showed that *Pp*GRXC5 was kinetically faster than *At*GRXC1 in the oxidative half-reaction (Bohle *et al*., 2024). Arabidopsis GRXC5 and poplar GRXS12 are both able to reduce the METHIONINE SULFOXIDE REDUCTASE B1 and PRXIIE (Gama *et al*., 2008; Tarrago *et al*., 2009; Couturier *et al*., 2011). Furthermore, poplar GRXS12 is able to deglutathionylate A_4_-GLYCERALDEHYDE-3-PHOSPHATE DEHYDROGENASE (GAPDH) and ISOCITRATE LYASE (Couturier *et al*., 2009b; Zaffagnini *et al*., 2012). Arabidopsis GRXC5 and GRXS12 were both found to deglutathionylate β-AMYLASE 1 (Gurrieri *et al*., 2025). This is consistent with the note that the first catalytic cysteine and the respective monothiol mechanism is sufficient for the reduction of protein disulfides (Zimmermann *et al*., 2020).

The only mutant described so far for the GRXC5 isoform is *P. patens Δgrxc5*. The null mutant has no obvious phenotype under standard growth or stress conditions induced by H_2_O_2_, heat, low light, and high light. *Δgrxc5*, however, showed a decreased sensitivity towards the thiol-specific oxidant DPS (2,2′-dipyridyl disulfide), indicating a disadvantageous GRXC5 activity under this condition in the wild type. To examine whether redox dynamics in the *Δgrxc5* mutant are affected, stromal-targeted roGFP2 was expressed (Bohle *et al*., 2024). Because GRXs catalyse the equilibration of roGFP2 with the local steady-state *E_GSH_*, roGFP2 is a suitable target protein providing a direct fluorescence read-out for the redox state (Meyer *et al*., 2007). Upon treatment with H_2_O_2_, the oxidation of roGFP2 proceeded similarly fast in the wild type and the *Δgrxc5* mutant. The recovery from the oxidative stress, monitored by reduction of roGFP2, was slower in the mutant, indicating that GRXC5 is important for GSH-dependent disulfide reduction (Bohle *et al*., 2024). In Arabidopsis, approximately 41% of glutathionylated proteins are present in the chloroplasts, indicating a plethora of putative target proteins for the GRXs (Müller-Schüssele *et al*., 2021). Reversible glutathionylation of a conserved C-terminal cysteine in TRXf has been proposed to play a key role in modulating TRX efficiency (Michelet *et al*., 2005). The number of glutathionylated target proteins may change in a light-dependent manner, because the stromal *E_GSH_* shows a pronounced light-responsiveness, particularly during light/dark and dark/light transitions (Müller-Schüssele *et al*., 2020; Haber *et al*., 2021). In the *P. patens Δgrxc5* mutant, light/dark-dependent changes of the roGFP2 redox state were absent, while in the wild type, the transition resulted in a rapid sensor oxidation followed by a recovery phase, indicating that GRXC5 is important for transmission of glutathione redox dynamics in the stroma to proteins (Bohle *et al*., 2024).

The impact of glutathionylation and stromal *E_GSH_* was recently shown for starch degradation in stomatal guard cells (Gurrieri *et al*., 2025). During dawn, starch is degraded in guard cells by the glucan hydrolases α-AMYLASE3 and β-AMYLASE1 (BAM1) to promote stomatal opening. It was shown *in vitro* that glutathionylation of BAM1 transiently protects the enzyme from H_2_O_2_-induced oxidation and concomitant formation of an intramolecular disulfide bridge that inhibits BAM1 activity. GRXC5 and GRXS12 were both able to deglutathionylate BAM1, pointing to a regulatory role of the plastidic GRXs in BAM1 activity. To explore the impact of plastidic glutathione homeostasis on BAM1 activity *in planta*, the authors investigated Arabidopsis mutants that have decreased activity of GR2, a glutathione disulfide reductase that is dual-targeted to plastids and mitochondria. The mutants showed higher BAM1 activity, lower starch levels in guard cells, and altered stomatal apertures, indicating that *E_GSH_* impacts stomatal physiology, possibly through BAM1 glutathionylation (Gurrieri *et al*., 2025). Whether the GRXs mediate the glutathionylation of BAM1 in response to the oxidative shift in the stromal glutathione pool was not shown.

Regarding GRXS12, a recent publication suggested that it acts as an oxidoreductase, reducing the Fe–S cluster assembly protein SUFB in the sulfur mobilization (SUF) pathway (Fig. 2C). This maintains proper Fe–S protein maturation, as well as chloroplast structure and photosynthetic efficiency (Liu *et al*., 2026). It was demonstrated that recombinant GRXS12 reduces oxidized SUFB, with a prominent redox-sensitive site mapped to C^415^. Reduced C^415^ is essential for acquiring sulfur from the cysteine desulfurase NFS2, and incorporating it into the Fe–S cluster. Consequently, in the *grxs12* null mutant, the oxidized fraction of SUFB increases, resulting in a reduction in the abundance of several chloroplast Fe–S proteins. Additionally, it was shown that, under oxidizing conditions, GRXS12 can form an intramolecular disulfide bond between the active-site cysteine C^34^ and a C-terminal C^92^ residue, which is also present in GRXC1 and GRXC2, as discussed above. This disulfide bond can be reduced by TRXm1, which further emphasizes the interaction between the two reducing systems (Liu *et al*., 2026). These results establish a novel, redox-dependent mechanism for regulating chloroplast Fe–S biogenesis, which is distinct from the Fe–S cluster transferase activity of class II GRXs, as will be discussed in the next section.

In summary, all Arabidopsis class I GRXs show efficient (de)glutathionylation as well as thiol-disulfide oxidoreductase activities *in vitro*. While some native substrates have been demonstrated, the full repertoire of physiological targets remains largely unknown. Other critical gaps remain for understanding localization-specific roles, clarifying the *in planta* significance of Fe–S cluster binding, and for integrating class I GRXs into broader plant signaling networks. Addressing these questions will provide deeper understanding of how plants maintain a redox balance in diverse cellular compartments and respond to dynamic environmental and metabolic challenges.

## Class II glutaredoxins act as chaperones for Fe–S cluster and unfolded proteins

The Arabidopsis genome contains four class II GRXs, which are localized to the mitochondria (1), to the cytosol (1), and to the plastids (2) (Figs 1, 3A). All four class II GRXs are defined by the fully conserved CGFS active site motif and a 5-amino acid insertion in the loop region preceding the α-helix 1 carrying the active site. The extended loop in the protein structure causes a repositioning of a strictly conserved lysine residue, which in class I GRXs electrostatically interacts with the C-terminal carboxyl group of the glycyl residue of GSH. The repositioning of the lysine residue in class II GRXs causes a relocation of the thiol group of GSH away from the active site, such that a rhombic [2Fe–2S] cluster can be ligated (Liedgens *et al*., 2020; Trnka *et al*., 2020). At the same time, relocation of the GSH thiol group severely affects the ability of class II GRXs to act as oxidoreductases. The ability to reduce roGFP2 as a disulfide target protein is completely lost, and the ability to oxidize roGFP2 with GSSG as an oxidant is largely diminished (Moseler *et al*., 2015; Begas *et al*., 2017; Liedgens *et al*., 2020; Trnka *et al*., 2020). However, deletion of an additional cysteine at the start of α-helix 3 close to the C-terminus enables oxidoreductase activities with roGFP2 as a substrate, albeit at lower activity than GRXC1 (Zannini *et al*., 2019). The predicted structure of the TRX-fold suggests that this C-terminal cysteine is positioned close to the active site, and indeed, intramolecular disulfide formation with the active site cysteine has been shown for *Chlamydomonas reinhardtii* GRX3 and Arabidopsis GRXS16 (Zaffagnini *et al*., 2008; Zannini *et al*., 2019). This disulfide cannot be reduced by GSH; rather, it requires reduced TRX (Zannini *et al*., 2019). However, the detailed molecular function of this disulfide is not yet understood, and the redox-related properties of class II GRXs have yet to be characterized in more detail. Despite this lack of knowledge, it is apparent that class II GRXs with increasing diversification have gained additional features which are linked to different functions in metabolism. These aspects will be discussed in the next sections.

**Fig. 3.**
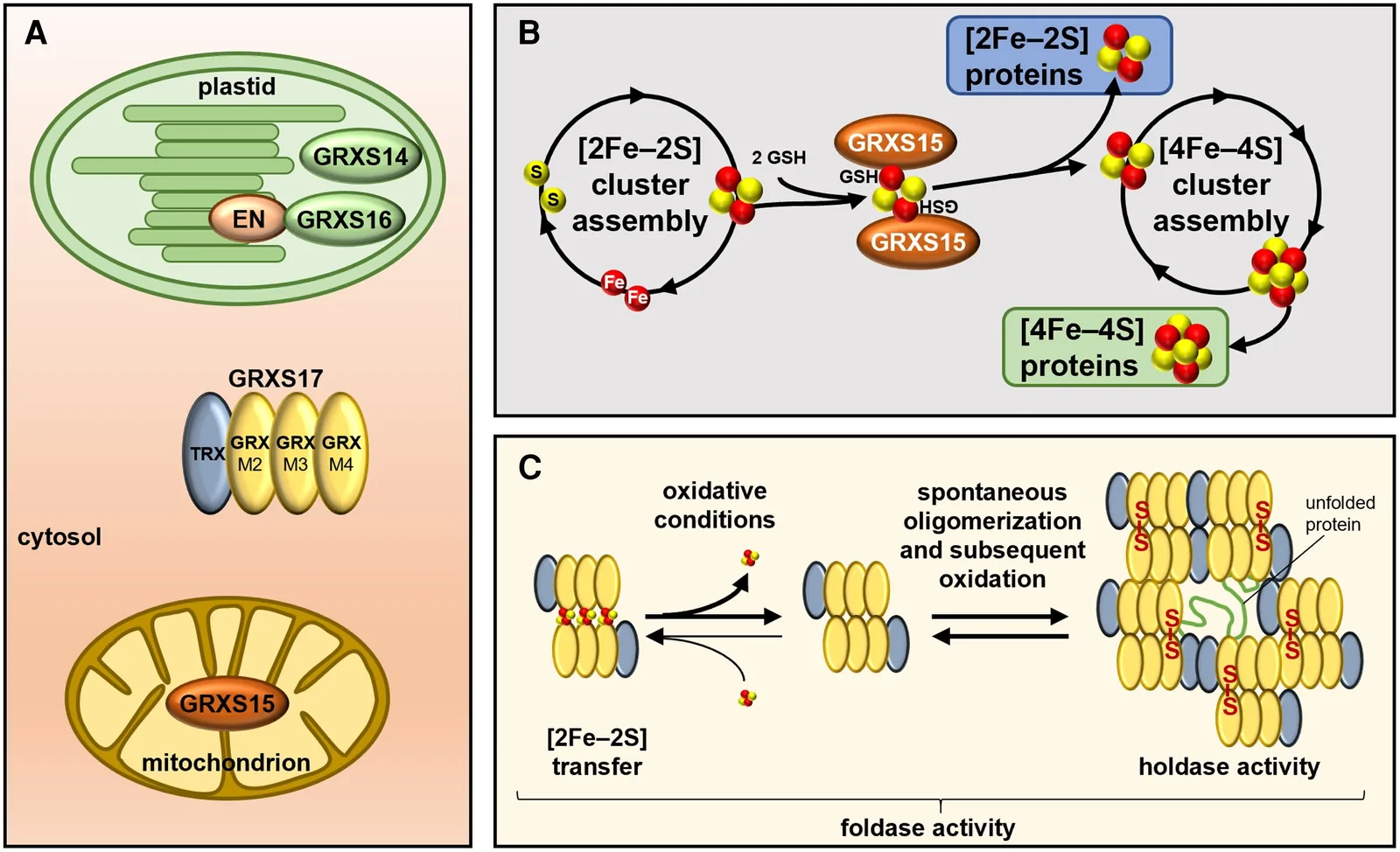
Sub-cellular localization and key functions of class II glutaredoxins. (A) Sub-cellular localization. All four class II GRXs from Arabidopsis are depicted as monomeric proteins. GRXS16 contains an additional N-terminal endonuclease (EN) domain. GRXS17 is a multidomain protein with one N-terminal TRX followed by three GRX domains, which are labelled as M2, M3, and M4 (Knuesting *et al*., 2015). (B) GRXS15 is involved in chaperoning and transferring [2Fe–2S] cluster in mitochondria. It is generally assumed that the other three class II GRXs fulfil similar functions in their respective compartments. (C) GRXS17 shows a foldase activity and an Fe–S cluster-dependent activity shift that converts the holoenzyme into a holdase involved in chaperoning proteins during heat stress. The depicted head-to-tail orientation for the dimer is based on a vague prediction from AlphaFold 3 (https://alphafoldserver.com/welcome). The organization of the oligomer and the exact positions of the disulfides is unknown und thus the depicted sketch is only for illustration of the principle.

### Mitochondrial GRXS15

The deletion of the mitochondrial GRXS15 in Arabidopsis is embryonic-lethal (Moseler *et al*., 2015), and is, in fact, the only confirmed lethal phenotype of any *grx* mutant described thus far. Like homologous class II GRXs in other species, GRXS15 is involved in the transfer of inherently unstable [2Fe–2S] clusters between their assembly machinery and the respective target proteins (Fig. 3B). This protects the cluster from deterioration and enables safe transfer. Interference with GSH coordination through substitution of a strictly conserved lysine residue (K^83^) in position –9 before the active site CGFS motif, generated a very weak *GRXS15^K83A^* allele with largely diminished Fe–S transfer capacity (Moseler *et al*., 2015). Complemented null mutants with the *GRXS15^K83A^* variant showed a severe growth phenotype similar to *grxs15* knock-down mutants (Moseler *et al*., 2015, 2021b; Ströher *et al*., 2016). Although the target proteins for the [2Fe–2S] cluster include a multitude of [2Fe–2S] cluster proteins and the assembly machinery for the cubane [4Fe–4S] cluster, most Fe–S cluster-dependent processes are surprisingly unaffected. These include biotin and molybdenum co-factor biosynthesis, the electron transport chain, and aconitase activity in the tricarboxylic acid cycle (Moseler *et al*., 2021b). Metabolic profiling of such mutants revealed pronounced increases only for pyruvate, 2-oxoglutarate, glycine and the breakdown products of the branched chain amino acids leucine, isoleucine and valine. These metabolites have one thing in common: their further use depends on four dehydrogenase complexes—pyruvate dehydrogenase, the 2-oxoglutarate dehydrogenase, the glycine decarboxylase, and the branched-chain α-keto acid dehydrogenase—all of which require lipoamide (6,8-dithioactonoic amide) as a prosthetic group. Lipoamide is synthesized by lipoyl synthase from a protein-bound octanoic acid by attaching two sulfur atoms to the carbon skeleton through radical reactions. For bacteria and human mitochondria, it has been shown that these two sulfur atoms originate from a [4Fe–4S] cluster (McCarthy and Booker, 2017; Warui *et al*., 2022). The manifestation of a phenotype resulting from severely limited [2Fe–2S] cluster transfer by GRXS15 is likely due to the combined effect of very low lipoyl synthase abundance and high demand resulting from the consumption of one [4Fe–4S] cluster, i.e. two [2Fe–2S] cluster per lipoyl co-factor (Pedroletti *et al*., 2023a, b). Indeed, a strong decrease of lipoamide-bound proteins was observed in *grxs15* knock-down lines (Ströher *et al*., 2016).

For transfer of [2Fe–2S] cluster to the [4Fe–4S] assembly machinery, GRXS15 physically interacts with iron-sulfur cluster A (ISCA)-type carrier proteins (Azam *et al*., 2020). Beyond this experimentally confirmed interaction, more interactions are likely, but are not confirmed yet. GRXS15 needs to interact with components of the [2Fe–2S] cluster assembly machinery, specifically with ISU/ISCU-like proteins, to pick up the newly assembled cluster. This assembly and transfer may need several co-chaperones, but so far evidence for direct interaction with GRXs is lacking [reviewed by Pedroletti *et al*. (2023a)].

Mitochondria typically maintain a highly negative *E*_GSH_ through action of a matrix-localized glutathione disulfide reductase (Schwarzländer *et al*., 2008; Marty *et al*., 2019). GRXS15 is the only GRX in Arabidopsis mitochondria, and therefore has been considered as a candidate for glutathionylation of roGFP2 as a target protein and possibly other targets. However, the activity for oxidation of roGFP2 *in vitro* is very low, and thus very unlikely to contribute significantly to specific protein thiol oxidation (Moseler *et al*., 2015; Begas *et al*., 2017). While the oxidoreductase activity of some class II GRXs can be enhanced by replacing the cysteine residue on α-helix 3 by serine, it should be noted that GRXS15 already carries a serine in the respective position. Following the same logic as applied in GRXS16 (see below), a serine-to-cysteine substitution in the respective position should further diminish the minute residual oxidation activity of GRXS15. However, this hypothesis has not yet been tested.

### Plastidic GRXS14 and GRXS16

The SUF pathway in plastids constitutes an independent Fe–S cluster assembly machinery (Balk and Schaedler, 2014). Transfer of assembled clusters to their respective recipient proteins requires a suitable protection system for the clusters, which is very likely performed by GRXS14 and GRXS16. Both *GRXS14* and *GRXS16* have been shown to complement a yeast *Δgrx5* mutant impaired in mitochondrial [2Fe–2S] transfer, which strongly argues for a conserved function (Bandyopadhyay *et al*., 2008). When grown under standard control conditions, a *grxs14* null mutant in Arabidopsis does not show any obvious phenotype. However, in combination with a knock down of *GRXS16*, plants were a bit smaller than the wild type controls (Rey *et al*., 2017). Although this argues for redundant function, the knockdown effect achieved in those experiments may not be sufficient to see phenotypes easily. Exposure of *grxs14* null mutants to darkness for eight days caused accelerated chlorophyll loss compared with the wild type. Because plants overexpressing *GRXS14* also showed a reduced chlorophyll content, the authors proposed that GRXS14 participates in the control of chlorophyll content in relation with light and osmotic conditions, possibly through a dual action in regulating the redox status of biosynthetic enzymes and contributing to the biogenesis of Fe–S clusters (Rey *et al*., 2017).

Like other class II GRXs, GRXS16 exhibits no activity in classical activity assays using small, non-protein compounds such as HED or DHA, and a glutathione recycling system. However, GRXS16 shows some thiol oxidase activity with roGFP2 as a dithiol target protein, which was much stronger than reported for GRXS15, but weaker than for GRXC1 as a class I standard control (Moseler *et al*., 2015; Zannini *et al*., 2019). A reducing activity could not be observed even with 10-fold excess of GRXS16, which is in line with the lack of such an activity in GRXS15. A particularly low p*K*_a_ of 3.9 for the active site cysteine C^158^ is consistent with the sensitivity of this residue to oxidation. A primary oxidation may be transmitted either to acceptor proteins like roGFP2, or, alternatively, be attacked by the additional cysteine C^215^ in the first position of α-helix 3. The resulting internal disulfide can only be reduced by TRX, but not by GSH (Zannini *et al*., 2019). With the competition between the formation of an internal disulfide and transfer of the primary oxidation to roGFP2, it is not surprising to find the roGFP2 oxidation significantly enhanced with a GRXS16^C215S^ mutant. More surprising is that the C^215^S substitution allowed GRXS16 to gain some reductive activity. This suggests that the more C-terminal C^215^, but not S^215^, interferes with the binding and activation of a GSH molecule for its attack on a target protein disulfide (Zannini *et al*., 2019; Geissel *et al*., 2024). While biochemically interesting, the physiological function of this partially conserved C-terminal cysteine is still far from being understood.

Arabidopsis GRXS16 shows another interesting feature in that it comprises an N-terminal domain with GlyIleTyr-TyrIleGly (GIY-YIG) endonuclease (EN) motif as a second functional domain (Fig. 3A; Liu *et al*. (2013)). The disulfide between C^62^ in the EN domain and C^158^ in the active site, which was originally proposed as a possible link between redox regulation and DNA metabolism in chloroplasts, could not be confirmed (Liu *et al*., 2013; Zannini *et al*., 2019). The physiological function of the EN domain remains unclear.

### Cytosolic GRXS17

Besides mitochondria and plastids, the cytosol also contains several Fe–S proteins for which an independent assembly machinery for Fe–S clusters is required. The biogenesis of Fe–S proteins in the cytosol and nucleus depends at least partially on mitochondria supplying precursors through ATP-BINDING CASSETTE TRANSPORTER OF THE MITOCHONDRIA (ATM) (Lill, 2009; Bernard *et al*., 2013; Zuo *et al*., 2017). In Arabidopsis, ATM3 has been proposed to export glutathione polysulfide, containing glutathione and persulfide, for cytosolic Fe–S cluster assembly (Schaedler *et al*., 2014). After the assembly of [2Fe–2S] clusters by the cytosolic Fe–S protein assembly (CIA) machinery, the labile clusters need to be protected from deterioration. Similar to other class II GRXs GRXS17 may act as a cytosolic Fe–S chaperone. However, GRXS17 differs from its mitochondrial and plastidic homologs, in that it is a multidomain protein consisting of an N-terminal TRX-like domain and three class II GRX domains, denominated M2, M3, and M4 (Fig. 3A). These domains all contain the highly conserved CGFS core motif, but differ in other parts, notably in the first position on α-helix 3, which is alanine in M2, serine in M3 and cysteine in M4. In analogy to the organellar class II GRXs, GRXS17 has been shown to coordinate three [2Fe–2S] clusters in the homodimeric form (Knuesting *et al*., 2015). However, despite the importance of Fe–S clusters as co-factors for multiple cytosolic and nuclear proteins, the deletion of *GRXS17* causes no strong phenotype under control conditions, and shows only a slightly impaired primary root growth (Cheng *et al*., 2011; Martins *et al*., 2020). The lack of a strong phenotype triggered doubts whether GRXS17 really acts as a chaperone during the transfer of [2Fe–2S] clusters (Knuesting *et al*., 2015). Nevertheless, interaction of GRXS17 with several proteins of the CIA complex and Fe–S proteins such as xanthine dehydrogenase was shown *in vitro* (Iñigo *et al*., 2016). Some class I GRXs can also coordinate a [2Fe–2S] cluster (Rouhier *et al*., 2007). However, these proteins fail to complement the yeast or zebrafish *Δgrx5* mutant (Bandyopadhyay *et al*., 2008; Trnka *et al*., 2020). Therefore, it remains an open question, which other proteins may provide protection for cytosolic [2Fe–2S] clusters during their transfer.

Deletion of *GRXS17*, however, was found to cause a pronounced phenotype when plants were exposed to heat and different light regimes. Null mutants grown at 28 °C displayed phenotypes consistent with defects in proliferation and/or cell cycle control while accumulating higher levels of ROS and cellular membrane damage (Cheng *et al*., 2011). When grown under long day conditions or even continuous light, the null mutants displayed elongated and thickened lamina and were delayed in their development (Knuesting *et al*., 2015). The *grxs17* null mutants also exhibited impaired auxin sensitivity and polar auxin transport, yet auxin biosynthesis remained unaffected. Consistent with the initial findings, the *grxs17* mutant showed an altered transcriptional response of many genes related to heat shock factors, auxin responses, cellular communication, and abiotic stress in comparison with wild type plants (Rao *et al*., 2023). GRXS17 has been proposed to interact with the NUCLEAR FACTOR Y SUBUNIT C11/NEGATIVE COFACTOR 2A (NF-YC11/NC2a), a nuclear transcriptional regulator that was isolated and confirmed in different interaction studies (Knuesting *et al*., 2015). Based on different independent data combining the heat- and light-dependent developmental phenotypes with the accumulation of ROS, and the observation that GRXS17 is prone to H_2_O_2_-mediated sulfenylation (Waszczak *et al*., 2014), a model has been proposed, according to which GRXS17 interacts with NF-YC11/NC2a to relay a redox signal generated by the photoperiod to maintain meristem function (Knuesting *et al*., 2015).

Deletion of *GRXS17* causes sensitivity to exogenous oxidants and iron deficiency stress. Conversely, ectopic expression of *AtGRXS17* enhances thermotolerance and confers chilling stress tolerance in tomato (Wu *et al*., 2012; Hu *et al*., 2015). This may be partially explained by a proposed protective role of GRXS17 in iron deficiency-induced oxidative damage (Yu *et al*., 2017). Based on alterations in the expression of iron-responsive genes in *grxs17* null mutants, it has been suggested that GRXS17 is necessary for tolerance to iron deficiency, and acts as a negative regulator under conditions of iron sufficiency (Cheng *et al*., 2020).

The release of the [2Fe–2S] clusters from GRXS17 dimers in response to a combination of high temperature with H_2_O_2_ also leads to a conformational change and the acquisition of holdase activities in the cluster-free form, which enables the binding of protein folding intermediates to prevent their aggregation (Fig. 3C; Martins *et al*. (2020)). These dimers can then spontaneously associate to oligomeric forms, and be stabilized through the formation of intermolecular disulfide bridges. GRXS17 apoproteins prevented the thermal aggregation of malate dehydrogenase and porcine heart mitochondrial citrate synthase, suggesting a pronounced holdase activity. Similarly, GRXS17 also exhibits an ATP-independent foldase activity that supports the refolding of denatured glucose-6-phosphate dehydrogenase. The foldase activity is exhibited irrespective of whether the Fe–S clusters are present or absent [Fig. 3C; Martins *et al*. (2020)]. Based on these results, the authors proposed that the Fe–S cluster enzyme GRXS17 is an essential guard that protects proteins against moderate heat stress. Although the foldase and holdase activities are not understood at the molecular level, the proposed model is consistent with genetic evidence from different plant species, which suggest a protective role of ectopically expressed *GRXS17* during thermal and drought stress (Wu *et al*., 2012, 2017).

In summary, all class II GRXs are capable of binding [2Fe–2S] clusters. This capability is exploited for chaperoning the clusters on their way from the primary assembly machineries to their respective target proteins in mitochondria, plastids, and the cytosol. Given that most Fe–S proteins are electron transfer proteins in energy metabolism or metabolic proteins, deficiencies in their [2Fe–2S] cluster binding will inevitably cause metabolic consequences. The same should apply to mutations in the protein-protein interaction sites that are involved in recognizing the [2Fe–2S] target proteins. However, no such mutations have yet been identified. Among class II GRXs, GRXS16 is special, due to its N-terminal EN domain, the function of which is not yet fully understood. What sets GRXS17 apart from other class members is that it contains three GRX domains and one TRX domain. This protein enlargement has enabled it to acquire the novel functions of a holdase and foldase, protecting proteins during high-temperature stress. Furthermore, GRXS17 is considered as an important regulator of the plant’s response to iron deficiency stress and the maintenance of redox homeostasis. However, the exact mechanisms underlying all these functions are not fully understood and require further detailed study.

## Class III glutaredoxins as transcriptional co-regulators

### Evolutionary history of class III GRXs

Class III GRXs, also known as ROXYs, probably evolved from the class I GRXs through diversification (Ziemann *et al*., 2009; Bodnar *et al*., 2023). As two ROXY isoforms are already present in bryophytes (e.g. *Marchantia polymorpha* and *P. patens*), the diversification of ROXYs mostly occurred in angiosperms, through paleopolyploidy events, and, more recently, via multiple tandem duplications (Ziemann *et al*., 2009). Phylogenetic analyses suggest that an original duplication in early land plants resulted in two distinct ROXY clades (A and B) (Fig. 1). A second duplication event occurred in clade A, forming two subgroups (Aα and Aβ), whose members are found in gymnosperms, basal gymnosperms, monocots, and eudicots. In clade B, an ancestral subgroup (Bα) is found in all land plants, with the notable exception of Arabidopsis, which may have lost such isoforms. The two angiosperm-specific Bβ and Bγ clades likely originate from two successive duplications, after which the Bγ subgroup has mostly expanded in monocot and eudicot branches (Ziemann *et al*., 2009; El Baidouri *et al*., 2024). The number of *ROXY* genes also varies greatly in both gymnosperms and angiosperms, ranging from 10 to over a 100 copies. For example, *H. syriacus,* has 145 paralogs. Notably, in the model plant Arabidopsis, 15 of the 21 isoforms are found within the Bγ subgroup. All ROXY members characterized so far are localized in the cytosol and nucleus (Fig. 1).

### Biochemical activity and transcription regulation

Class III GRXs are called CC-type because they contain a conserved CC(M/L)C or CC(M/L)S active site motif, rather than the C(G/P/S)Y(C/S) motif found in class I, or the CGFS motif found in class II (Rouhier *et al*., 2008). Biochemical studies conducted on some of these members have revealed only weak or no oxidoreductase activity on typical GRX substrates such as HED or roGFP2 (Xu *et al*., 2022; Mrozek *et al*., 2025). Recently, Mrozek *et al*. (2025) showed that the lack of oxidoreductase activity of ROXY9 on model substrates is due to the low reactivity of its active site cysteine for S-glutathionylation, which is a prerequisite for enzymatic activity. Replacement of the ROXY9 CCLC motif by a class I motif CPYC does not resume oxidoreductase activities on HED and roGFP2. On the other hand, ROXY9 shows a weak S-deglutathionylation activity on GAPDH (Zaffagnini *et al*., 2008; Mrozek *et al*., 2025), which might indicate a selectivity of reduction activity, depending on the substrate. Again, replacing the CCLC motif of ROXY9 with CPYC or CCLS did not alter its GAPDH-reducing activity. Therefore, rather than being dependent on the CCLC motif, it was proposed that structural alterations in the GSH binding site, which lead to an altered GSH binding mode through the induced fit of specific substrates, enable enzymatic activity and may explain enzymatic inactivity (Mrozek *et al*., 2025).

The ability of ROXYs to interact with bZIP-type TGA transcription factors has been demonstrated for members of different ROXY clades, using yeast two-hybrid (Y2H) or fluorescence complementation methods (Ndamukong *et al*., 2007; Li *et al*., 2009; Murmu *et al*., 2010; Gutsche *et al*., 2017; Ruan *et al*., 2018), and this interaction occurs mainly in the nucleus. However, the exact nature of the ROXY/TGA interaction is still being debated. For example, ROXY1 preferentially interacts with TGA2, TGA3, TGA7, TGA9, TGA10, and PERIANTHIA (PAN), but shows low affinity for TGA1 and TGA4–6 (Li *et al*., 2009; Murmu *et al*., 2010). In contrast, ROXY8, ROXY9, ROXY18, and ROXY19 interact more efficiently with TGAs 1–6 (Uhrig *et al*., 2017; Li *et al*., 2019), suggesting a certain degree of interaction specificity between ROXY and TGA members. Y2H and *in planta* studies have shown that a 14-aa domain in the C-terminus of ROXY1, containing an α-helical LxxLL motif, is necessary for interaction with TGAs (Li *et al*., 2009, 2011; Gutsche *et al*., 2017). In this same C-terminal domain, some ROXY members contain a terminal ALWL sequence that is involved in interaction with the transcriptional co-repressors TOPLESS and TOPLESS-RELATED (Uhrig *et al*., 2017). These partners can form a ternary complex, and it has been shown that interaction of ROXY with TGA is necessary for the repressive role of the ternary complex on the expression of target genes, such as *ROXY18* and *19* themselves (La Camera *et al*., 2011; Uhrig *et al*., 2017). Interestingly, 3D-based co-localization methods have revealed the co-localization of ROXY1 with various RNA polymerase II (RNAPII) isoforms that are active at different stages of the transcription cycle. This suggests that ROXY1 is necessary for initiation of transcription, but also plays a role in subsequent processes until the final stages of termination (Maß *et al*., 2020).

Various genetic analyses have revealed the functional nature of the interaction between ROXYs and TGAs. The role of the CCM/LC/S motif of ROXY has also been documented, although the mechanism of action remains to be clarified. For example, ROXY1 relieves the repressive activity of the TGA transcription factor PAN in the initiation of petal development (Li *et al*., 2009). Mutation of the first cysteine (C^49^) of the ^49^CCMC^52^ motif abolishes the complementation of the *roxy1* mutant phenotype, unlike mutation of C^52^, pointing to the key role of the first cysteine of the motif for the biological function of ROXY1 (Xing *et al*., 2005). The maize CC-type GRX MALE STERILE CONVERTED ANTHER1 (MSCA1) negatively regulates the activity of the TGA transcription factor FASCIATED EAR 4 (FEA4) in controlling meristematic activity (Yang *et al*., 2021). The native MSCA1 protein, but not a SCMS variant of the CCMC motif, is able to reduce the oxidized dimeric form of FEA4 involving the conserved C^321^ of FEA4, thereby affecting DNA accessibility and transcriptional activity of FEA4 (Yang *et al*., 2021) (Fig. 4A). Nevertheless, the authors did not directly show the role of the cysteines in the CCMC motif in complementing the *msca1* mutant. Converting the CCMC motif to CPYS or CGFS in ROXY19 does not affect its transcriptional repressor activity, in contrast to an SSMS variant, which again highlights the role of the first cysteine of the CCMC motif for ROXY function (Zander *et al*., 2012; Huang *et al*., 2016).

**Fig. 4.**
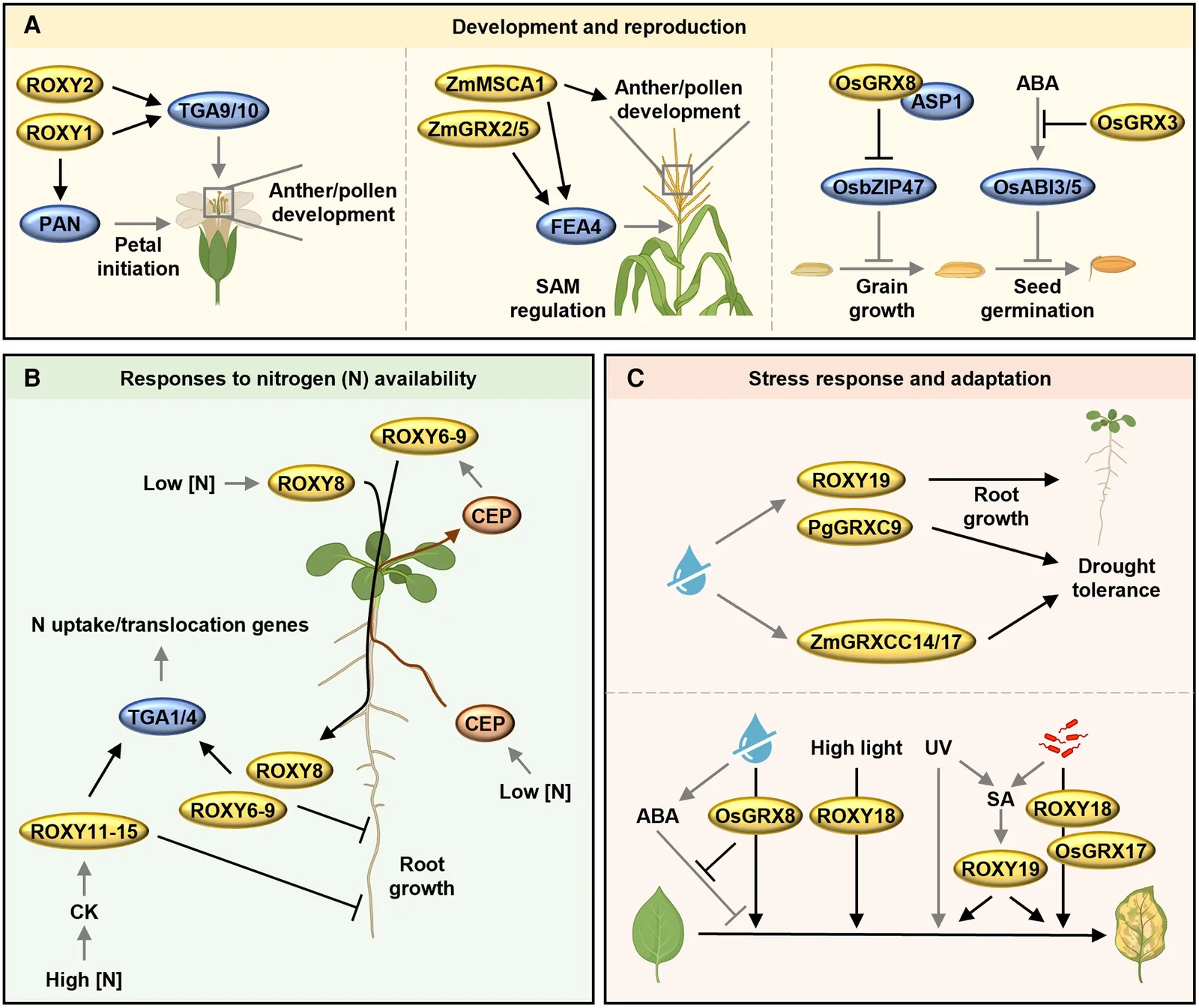
Key functions of class III glutaredoxins. Functions are categorized as reported, according to their role in (A) development and reproduction, (B) responses to nitrogen availability or (C) stress response and adaptation. (A) ROXY1/2 regulate anther/pollen development and petal initiation by activating the transcription factors TGA9/10 and PAN, respectively (left panel). In maize, MSCA1 and GRX2/5 regulate anther/pollen development and the shoot apical meristem (SAM) by activating the transcription factor FEA4 (middle panel). In rice, GRX8 and GRX3 regulate grain growth and seed germination by inhibiting the ABA-dependent ABI3/5 transcription pathway and the transcription factor ZIP47, respectively (right panel). (B) Under low and high nitrogen conditions, respectively, ROXY6-9 and ROXY11-15 regulate genes involved in N uptake and translocation by activating the transcription factors TGA1/4, while inhibiting root growth. (C) Under water deficiency, PgGRXC9, ROXY19, and ZmGRXCC14/17 regulate root growth and drought tolerance (upper panel). OsGRX8, OsGRX17, ROXY18 and ROXY19 regulate various responses to biotic and abiotic stress. Arabidopsis isoforms are indicated by the corresponding ROXY isoform names. For ROXYs from other plant species, the nomenclature is shown as described in the literature with prefixes to identify the corresponding species (Os: *Oryza sativa*; Zm: *Zea mays*; Pg: *Pennisetum glaucum*). ABA, abscisic acid; ABI3/5, transcription factors ABA INSENSITIVE 3 and 5; CEP, C-terminally encoded peptide; FEA4, transcription factor FASCIATED EAR 4; PAN, transcription factor PERIANTHIA; SA, salicylic acid; SAM, shoot apical meristem; UV, ultraviolet light.

### Development and reproduction

The role of specific ROXY isoforms in different aspects of plant development has been documented (Fig. 4A). In angiosperms, several ROXYs from clades Bα and Bβ control flower development and male fertility. In Arabidopsis, a member of the clade Bβ, ROXY1, controls petal development. The *roxy1* mutant displays a lack of petal primordia initiation and abnormal petal shape (Xing *et al*., 2005). In addition, the *roxy1 roxy2* double mutant is sterile and does not produce pollen due to perturbed anther development. This function is dependent on the glutathione-binding activity of ROXY1, and is likely to involve interaction with TGA9 and TGA10 (Xing and Zachgo, 2008; Murmu *et al*., 2010). In maize, clade Bα isoform MSCA1 controls cell fate in developing anthers (Chaubal *et al*., 2003). The *msca1* mutant produces vascular tissue instead of archesporial cells at the center of the anther. Because decreasing oxygen or H_2_O_2_ rescues germinal differentiation in *msca1* flowers, it has been proposed that MSCA1 acts as a redox regulator that senses the local redox status during germ cell specification (Kelliher and Walbot, 2012). Similar male sterility phenotypes were shown in a rice mutant of the closest homolog of *MSCA1*, *OsGRX19/MIL1*, as well as in a mutant lacking *OsGRX6/GRX_I1* (Hong *et al*., 2012; Yang *et al*., 2016). In both cases, the mutant phenotype was caused by defects in the entry of sporogenous cells into meiosis. In maize, *MSCA1* plays an additional role in the shoot apical meristem and phyllotaxis, due to modified PIN1-dependent auxin distribution prior to primordia development. Here, MSCA1 acts redundantly with its two closest paralogs from clade Bβ, ZmGRX2 and ZmGRX5, to regulate the activity of FEA4 (Yang *et al*., 2015, 2021) (Fig. 4A). In Arabidopsis, ROXY8 and ROXY9 (clade Bγ) regulate leaf hyponastic growth triggered by high/low light transitions. This regulation presumably occurs through repression of clade II TGAs, TGA1 and TGA4. However, ROXY19 and TGA2/5/6 are involved in salicylic acid-mediated inhibition of hyponastic growth (Li *et al*., 2019). In rice, OsGRX3 (clade Aβ) was identified as a negative regulator of abscisic acid (ABA) signaling during dormancy and seed germination, due to its ability to interact with the partner proteins of ABA receptors (Fig. 4A). An *OsGRX3* gain-of-function allele exhibits pre-harvest sprouting phenotype, and defects in stored seed viability, while a *Osgrx3* allelic null mutant displays delayed germination (Xu *et al*., 2019). Moreover, OsGRX8 (also called WG1), a member of clade Bβ, promotes rice grain growth by restricting the transcriptional activity of OsbZIP47 by associating with the transcriptional co-repressor ABBERANT SPIKELET AND PANICLE1 (ASP1), and induces cell proliferation. (Hao *et al*., 2021). In a related study, OsGRX8/WG1 was shown to modulate the oligomerization state of a guanine nucleotide-binding protein (G protein) SUBUNIT3 (GS3), alleviating its inhibitory effect on the interaction between RICE Gβ SUBUNIT1 (RGB1) and two Gγ SUBUNITS (DEP1/GGC2), and resulting in an increase in grain length. Here, an oxidoreductase activity of OsGRX8/WG1 was reported to reduce disulfide bonds in oligomeric GS3 complexes (Fig. 4A) (Liu *et al*., 2025).

### Responses to nitrogen availability

The importance of ROXYs in modulating root development is further emphasized by their involvement in the metabolic responses to nitrogen (N) availability, which affects the root system architecture (RSA) (Fig. 4B). Under high N conditions, lateral root development is suppressed once the plant has met its N demand. This is to prevent the plant from absorbing excess N that it cannot use. The underlying signaling pathway is driven by the drought-related phytohormone ABA (Araus *et al*., 2020). Several genes from the Bγ clade that lack the ALWL C-terminal motif (*ROXY11–15*, as well as the closely related *ROXY4*, *ROXY16*, and *ROXY17*) are rapidly up-regulated under high N conditions. Moreover, RNAi lines compromised in *ROXY11–13,15* expression display enhanced primary root growth, which supports the model that members of the ROXY11–15 cluster act as negative regulators of primary root growth, and therefore integrate plant N availability with root development (Patterson *et al*., 2016; Guo *et al*., 2017). Further investigations have shown that *ROXY12* and *ROXY15* are expressed in root and shoot vasculature in a nitrate-dependent manner. This regulates nitrate uptake and translocation to shoots by inducing the expression of the major nitrate transporter-encoding genes *NRT1.1*, *NRT2.1*, *NRT2.2*, and *NRT3.2* (Ehrary *et al*., 2020). In Arabidopsis, a systemic N acquisition response is triggered by the root-to-shoot mobile peptide hormone C-TERMINALLY ENCODED PEPTIDE (CEP) upon N starvation (Fig. 4B). CEP originates from the N-starved roots and induces the expression of ROXY6/CEPD1, ROXY7, ROXY8 and ROXY9/CEPD2, which all belong to the clade Bγ. ROXY6/CEPD1 and ROXY9/CEPD2 have been identified as mobile, shoot-to-root signals that induce the expression of the high affinity nitrate transporter *NRT2.1* only in root parts that are exposed to nitrate (Ohkubo *et al*., 2017). In addition, the closely related ROXY8/CEPDL2 also functions as a phloem-mobile signal that controls N uptake in response to the N status in the shoot (Ota *et al*., 2020). Recently, a *roxy6 roxy7 roxy8 roxy9* quadruple mutant was shown to be impaired in the transcriptional activation of N-starvation-inducible genes, most likely due to the association of clade I TGAs with a repressive complex at their target promoters (Thurow *et al*., 2025). It was also suggested that ROXY6–9 and ROXY10–17 bind competitively to TGA1 and TGA4 in the roots (Fig. 4B). The former act as transcription co-activators, enhancing N uptake, while the latter function as co-repressor complexes together with TOPLESS (TPL), TPL-related1 (TPR1) and TPR4, limiting N uptake (Kobayashi *et al*., 2024). Two other studies showed that *ROXY6/CEPD1* and *ROXY9/CEPD2* also integrate the systemic CEP-derived signal and the local cytokinin levels in the root, to fine tune the RSA in response to N availability (Taleski *et al*., 2023; Chapman *et al*., 2024). Overall, these studies support the idea that Arabidopsis clade Bγ *ROXY*s play a complementary role in adapting the RSA to the N status of the plant.

### Stress response and adaptation

The responsiveness of ROXYs to plant hormones has been well documented. In Arabidopsis, *ROXY19* expression is highly induced by SA, in a NON-EXPRESSOR OF PR GENES 1 (NPR1) and TGA-dependent manner (Fig. 4C). It has been proposed that ROXY19, TGA2, and NPR1 form a ternary complex to control the expression of SA- and jasmonate (JA)-dependent responses (Ndamukong *et al*., 2007; Zander *et al*., 2012). The *ROXY19* gene is also induced by UV-B via an SA-dependent but NPR1-independent mechanism (Herrera-Vasquez *et al*., 2015). This latter induction is dependent on binding of TGA2/3 to *as1-like cis*-elements in the *ROXY19* promoter. Conversely, ROXY19 binds to the TGA2/3 complex on its own promoter, attenuating its own transcription. ROXY19 also interferes with the activation of TGA-dependent detoxification genes, resulting in an increased sensitivity to xenobiotic stress (Huang *et al*., 2016).

The response to infection with pathogens like *Botrytis cinerea* is mediated by ROXYs. In Arabidopsis, rice, and cucumber, *AtROXY1*, *AtROXY18, OsROXY1*, and *CsGRX4* are all transcriptionally induced in response to infection via TGA and biotic stress-related hormonal pathways (JA, SA, ethylene). However, these ROXYs generally act as negative regulators of plant defense responses to *B. cinerea*, facilitating infection (Wang *et al*., 2009; La Camera *et al*., 2011; Yang *et al*., 2022). The closely related OsGRX17 in rice also negatively regulates disease resistance by repressing the transcription of *OsSGT1*, a critical component of SA inactivation, and subsequent defense genes, via interaction with rice TGA-like transcription factor1 (OsTGAL1) (Li *et al*., 2022) (Fig. 4C).

Finally, ROXYs are involved in responses to various abiotic stresses. ROXY18 acts by limiting methyl viologen or high light-induced photooxidative stress (Laporte *et al*., 2012). Rice OsGRX8, a close homolog of Arabidopsis ROXY1 and ROXY2, which is induced by drought, oxidative stress, and auxin, functions as a negative regulator of hormone and stress responses. Overexpression lines display hyposensitivity to hormones (auxin, ABA) and stress (salt or oxidative stress) treatments, whereas RNAi lines show opposite hypersensitive phenotypes (Sharma *et al*., 2013). In cassava, six *ROXY* genes (clade Bγ *MeGRXC3, MeGRXC4, MeGRXC7, MeGRXC14, MeGRXC15*, and clade Aβ *MeGRXC18)* are induced upon water deprivation, and in response to ABA (Ruan *et al*., 2018; Guo *et al*., 2022). In rice, OsGRX4, which belongs to the Bγ clade, is involved in the response of both shoots and roots to salt treatment, as well as to mannitol or H_2_O_2_ (Verma *et al*., 2021). In both maize and pearl millet, natural polymorphisms in ZmGRXCC14 and PgGRXC9 are associated with drought tolerance through their control of primary root growth (Ding *et al*., 2019; de la Fuente *et al*., 2024) (Fig. 4C). Additionally, the closest homolog of PgGRXC9 in Arabidopsis, ROXY19, also regulates primary root growth through the control of cell elongation (de la Fuente *et al*., 2024) (Fig. 4C).

In summary, class III GRXs are specific to land plants, and the number of their genes has increased significantly over the course of evolution through various duplication events. This expansion has enabled the diversification of the functions performed by its members during different stages of plant development and reproduction, nitrogen metabolism, and responses to various biotic and abiotic stresses. These responses are primarily associated with the nuclear functions of ROXYs, which act as transcriptional regulators due to their ability to interact with transcription factors and transcriptional co-regulators. While a few examples suggest weak deglutathionylation activity and the reduction of disulfide bridges, the underlying mechanism of this activity remains poorly documented in most cases.

## Class IV glutaredoxins

The two members of the GRX class IV, GRXlike1 and GRXlike2, have only been identified based on genomic information. Both predicted proteins contain a GRX domain and, at a length of ∼630 amino acids, are much larger than typical GRXs, which contain only 120–150 amino acids. The increased length is based on the fusion to two other domains, a putative DEP (Dishevelled, Egl-10 and Pleckstrin domain) and DUF547 (Domain of Unknown Function 547) domain (Couturier *et al*., 2009a). Both proteins are predicted to be localized in the cytosol. Publicly accessible databases indicate that both genes are expressed at very low levels, with limited response to abiotic and biotic stresses (https://bar.utoronto.ca/efp//cgi-bin/efpWeb.cgi). So far, biochemical characterization of these proteins has been precluded by an inability to purify full-length recombinant proteins. DEP domains are known to be involved in G-protein-dependent signaling pathways, and have been proposed to be responsible for selective targeting of the respective proteins to distinct membrane sites (Ballon *et al*., 2006; Tang *et al*., 2023). The presence of DEP domains in class IV GRXs may thus offer a lead for further functional characterization.

## Concluding remarks

Land plants diverged from green algae around 475 million years ago and subsequently led to the major classes and families found today. The corresponding plants exhibit distinct adaptations to diverse ecological niches and concomitant abiotic and biotic challenges, which correlate with the diversification of GRXs arising from genome duplication events and subsequent selection of adaptive traits. The most pronounced diversification is evident in the plant-specific class III ROXY-type GRXs. These have already split into five sub-classes, four of which are present in Arabidopsis (Fig. 1). Current knowledge suggests that these sub-classes are associated with various functions, including developmental processes and responses to biotic and abiotic stresses (Fig. 4). All these functions are characterized by the role of ROXYs as transcriptional co-regulators. However, the limited mechanistic evidence for these regulatory principles, coupled with the potential for redundancy, suggest that the precise role of ROXYs remains unclear.

Diversification has also occurred in class I GRXs, which exhibit pronounced oxidoreductase activities. In this case, diversification occurred with the transfer of class I GRXs to plastids and the ER. In addition, the cytosolic GRXC1 evolved as a new member of class I GRXs only in eudicots. Despite their confirmed biochemical activities, the exact physiological role of these GRXs remains unknown. The main challenge in conducting functional analyses is that null mutants do not exhibit significant phenotypes. This may suggest that class I GRXs mainly fine-tune other proteins through redox-dependent post-translational modification. Plants have a huge diversity of oxidoreductases in the TRX superfamily, so they may well have maintained partially redundant activities that prevent phenotypes from occurring. Interestingly, plant mitochondria do not have class I GRXs, so the question remains as to whether other oxidoreductases have taken over the respective functions.

Class II GRXs are more conserved, even across biological kingdoms. The near-perfect conservation of the CGFS active site suggests that the associated physiological process is one of the oldest and most essential. Indeed, the protected transfer of labile [2Fe–2S] clusters to their target proteins is essential in the cytosol, mitochondria, and plastids. Considering that the Last Universal Common Ancestor already contained two distinct ‘minimal’ Fe–S cluster assembly machineries, it will be interesting to analyse when GRXs were integrated into this machinery and likely enhanced biogenesis and distribution of Fe–S cluster (Garcia *et al*., 2022).

However, evolution has brought about further changes that are still not fully understood. In the cytosol, GRXS17 has undergone domain multiplication, including an additional TRX domain, which may be linked to the acquisition of novel functions. Recent research has revealed that GRXS17 acts as a chaperone in plants exposed to heat stress (Martins *et al*., 2020). The molecular mechanism of this function requires further analysis. Meanwhile, the evolutionarily older Fe–S transfer in the cytosol appears to be at least partly taken over by other proteins, whose identities are yet to be determined. In the plastids, acquisition by GRXS16 of an EN domain has led to speculation about a potential connection between redox homeostasis and DNA metabolism (Liu *et al*., 2013). However, the function of the EN domain remains unknown, and the proposed mechanism for redox control has been disproven (Zannini *et al*., 2019). In general, biochemical evidence suggests limited oxidoreductase activities of class II GRXs, but it remains virtually unknown whether these activities are relevant to metabolism. In this context, there is still an open question on the physiological relevance of a partially conserved cysteine residue on α-helix 3 in class I and class II GRXs.

Biochemical studies also suggest a limited oxidoreductase activity for class III GRX members. Although a low deglutathionylation activity has been described for ROXY9, and other members seem to exhibit a disulfide bond reduction activity, recent biochemical data on ROXY9 suggest that its weak oxidoreductase activity is specific to this class of GRX due to its structural characteristics (Mrozek *et al*., 2025). Nevertheless, not all the protein partners of ROXYs are known. A more specific enzymatic activity of ROXYs could focus on target proteins that are still unknown. The role of ROXYs in nuclear processes as transcriptional regulators has been well documented over the past decade. In contrast, the function of the cytosolic localization of most ROXYs remains completely unexplored. Similarly, *in silico* data indicating that some ROXY members (e.g. ROXY11) may be localized within the secretory pathway could stimulate new research into the metabolic functions of ROXYs. Likewise, the tissue localization and response to environmental stresses of ROXYs have received little documentation so far (Belin *et al*., 2015). Further studies of expression patterns could partly explain the diversification, given that many members are currently considered redundant, in terms of their functions in nitrogen metabolism, for example.

Overall, there is overwhelming evidence that GRXs play an important role in plant metabolism. However, the specific mechanistic details are still unclear. A key difference between plants and non-plant species is the high degree of redundancy resulting from multiple genome duplications and incomplete functional diversification. This is a limiting factor in research on plant GRXs. However, with the ability to generate new mutants and, in particular, multiple mutant combinations through targeted genome editing, it is possible to gradually overcome this limitation and provide new mechanistic insights.
